# Gamifying Self-Management of Chronic Illnesses: A Mixed-Methods Study

**DOI:** 10.2196/games.5943

**Published:** 2016-09-09

**Authors:** Alaa AlMarshedi, Gary Wills, Ashok Ranchhod

**Affiliations:** ^1^ University of Southampton School of Electronics and Computer Science Southampton United Kingdom; ^2^ University of Southampton Winchester School of Art Winchester United Kingdom

**Keywords:** gamification, healthcare, self-management, chronic illnesses, diabetes, motivation, behavioral change

## Abstract

**Background:**

Self-management of chronic illnesses is an ongoing issue in health care research. Gamification is a concept that arose in the field of computer science and has been borrowed by many other disciplines. It is perceived by many that gamification can improve the self-management experience of people with chronic illnesses. This paper discusses the validation of a framework (called The Wheel of Sukr) that was introduced to achieve this goal.

**Objective:**

This research aims to (1) discuss a gamification framework targeting the self-management of chronic illnesses and (2) validate the framework by diabetic patients, medical professionals, and game experts.

**Methods:**

A mixed-method approach was used to validate the framework. Expert interviews (N=8) were conducted in order to validate the themes of the framework. Additionally, diabetic participants completed a questionnaire (N=42) in order to measure their attitudes toward the themes of the framework.

**Results:**

The results provide a validation of the framework. This indicates that gamification might improve the self-management of chronic illnesses, such as diabetes. Namely, the eight themes in the Wheel of Sukr (fun, esteem, socializing, self-management, self-representation, motivation, growth, sustainability) were perceived positively by 71% (30/42) of the participants with *P* value <.001.

**Conclusions:**

In this research, both the interviews and the questionnaire yielded positive results that validate the framework (The Wheel of Sukr). Generally, this study indicates an overall acceptance of the notion of gamification in the self-management of diabetes.

## Introduction

The health care industry is experiencing significant changes due to advances in Health 2.0 and mobile technologies [1]. A more user-centered approach is being used to facilitate change in health provision. There has been a focus on utilizing mobile technologies for health behavior interventions. Mobile technologies provide a medium to easily connect to patients and create change. Moreover, apps can be used to track medication, manage illness, and monitor health. Also, online communities can provide patients with the emotional and psychological support they need. However, some Web and mobile health care interventions in their current form lack effective and engaging qualities; they may not appeal to a lot of patients and their effect is temporary.

Gamification could be the solution to effective health care interventions. It is a concept that borrows from game techniques but is not a game by itself. A number of game elements, including badges, levels, leader boards, and progress bars are used in gamification [2]. The principles of engagement, reward, and incentive are incorporated in certain tasks to encourage changes in behavior or motivate users to learn new skills [3]. Gamification employs the innate urge for recognition and instant positive feedback as a way to promote change in behavior and drive user engagement. Moreover, rewards typically leave people feeling happy [4].

Gamification can be thought of as a motivational tool [5]. Generally, motivation can occur in many ways to achieve goals, satisfy personal needs, fulfill leader expectations, and gain rewards or incentives [6]. The health care community is now realizing the power of gamification on motivation [7-10]. Thus, some gamification features have been incorporated in a number of health and fitness apps [11,12]. SuperBetter is one of the successful examples of gamification. It is a tool for self-improvement that provides users with an engaging and interactive experiment to help them reach their health goals [13]. Gamification has been also incorporated in some self-management apps for diabetic patients such as MySugr [14]. MySugr helps users track their blood glucose levels along with other relevant data such as their carbohydrate intake and medication. The app also allows users to keep a photo diary of their meals. Furthermore, it rewards users who are committed to logging their information. However, the app is limited by geographical location as it is available only to users in the United States and Europe. However, the gamified apps for self-management do not follow a specific framework or guideline, and there has been relatively little research on the use of gamification in self-management and adherence to medication.

Chronic illnesses such as diabetes could benefit from the use of gamification [15]. Diabetes is considered the “disease of the 21st century,” and the most common chronic illness in the world [16]. Encouraging adherence to medication and self-management is crucial for the health of a diabetic person. Having diabetes requires a great deal of self-care, such as taking medication, keeping track of food intake, and exercising. It requires self-management skills that are vital in preventing the complications associated with the disease and maintaining a healthy life [17]. This includes the ability to deal with diabetes requirements such as lifestyle changes, medication, and physical and social consequences. Mobile apps can help patients self-manage in a more efficient manner [18].

Gamification has the potential to positively influence patients with chronic illnesses in adhering to medication and self-managing more effectively [15,19]. It can make the tedious and repetitive tasks of managing a chronic illness such as diabetes rewarding and more engaging [12]. Moreover, it can lead to an increase in the adoption of digital health care services, which is generally slow, often because such services are poorly designed and do not meet user needs [20]. In a recent study, 75% of participants showed interest in using digital health services, especially if they provide assistance with routine health tasks [20]. However, the sole reliance on points and badges could damage the longevity effect of gamification and thus diminish the purpose of gamification in the first place. While points and badges are a part of gamification, there are other crucial game techniques that need to be considered. Therefore, to benefit from all the advantages of gamification, one needs to understand the environment to which it is applied, so specific gamification techniques can be tailored and applied to this specific environment.

To address this, we introduced a framework named the Wheel of Sukr [21]. To our knowledge, this is the first framework that targets the use of gamification in the self-management of chronic illnesses (more details of the design rationale are found in [21]). It combines game elements, self-management practices, and behavioral change methods to provide effective and better self-management systems that reinforce healthier behavior. The framework addresses the issues of engagement and effectiveness of self-management applications. It also turns the self-management “tasks” into fun and rewarding activities.

**Figure 1:**
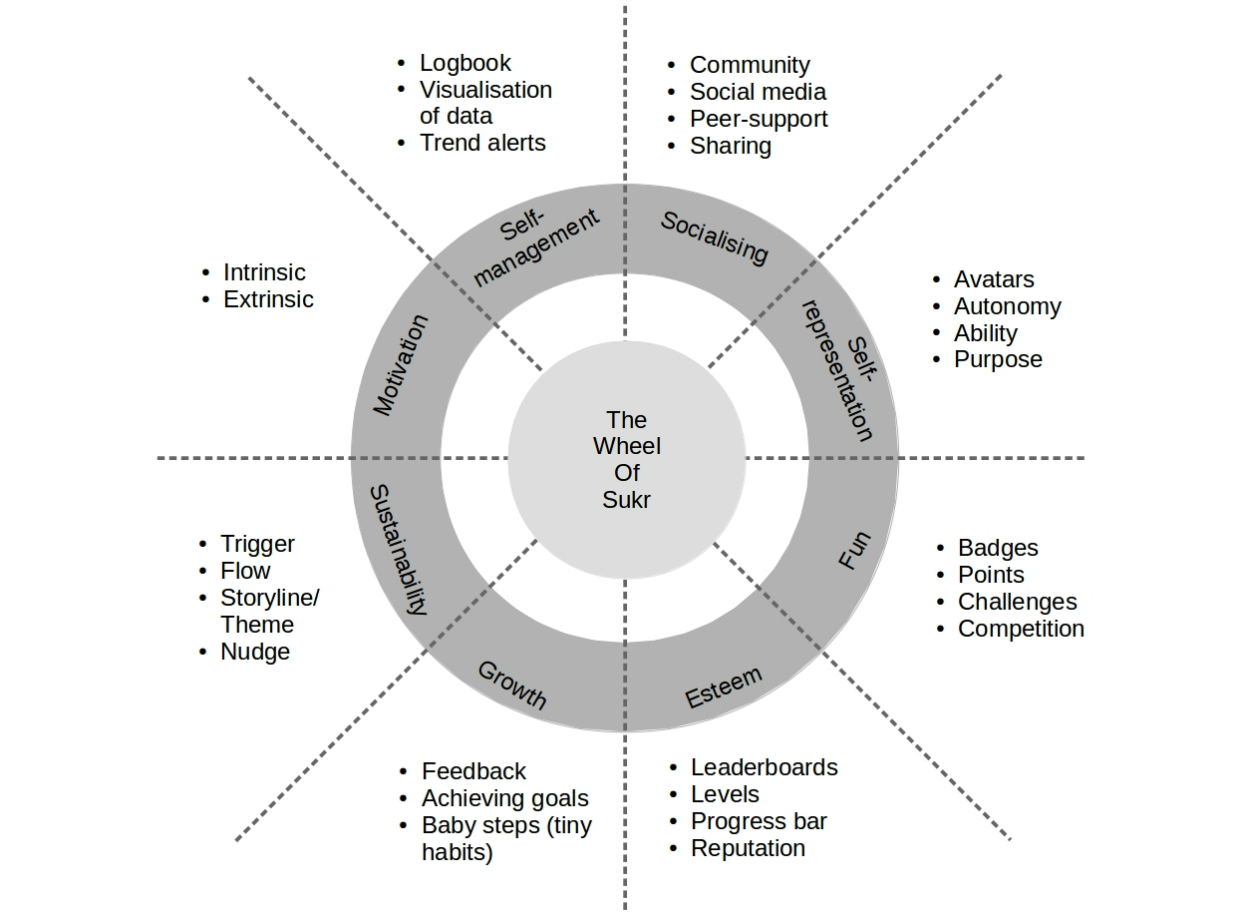
The Wheel of Sukr.

The Wheel of Sukr (Figure 1) contains 28 elements organized under eight different themes:

1. Self-management [22,23]: Basic elements needed to self-monitor blood glucose, including tracking measures of blood glucose, insulin, food intake, and other related information; getting feedback based on the entries; and being notified when blood glucose measures fluctuate.

2. Socializing [24,25]: Being part of a group of people that shares the same situation, which offers social and emotional support and adds to the value of rewards.

3. Self-representation [26,27]: Tailoring the experience to the user to create a bond with the user, thus, increasing engagement and resulting in a meaningful experience.

4. Fun [2,28]: Creating a game-like experience.

5. Esteem [29,30]: Satisfying the fourth level of Maslow’s Hierarchy of Needs, thus, catering to the psychological side of managing diabetes.

6. Motivation [31]: Appealing to the desire to do things.

7. Sustainability [27,32]: Maintaining the same level of engagement to sustain the desired effect.

8. Growth [33]: Creating a fruitful experience for the user, where gamification in a social and psychological context can result in personal growth in terms of managing diabetes, learning new healthy habits, and understanding the disease better.

In this paper, we present a validation of the Wheel of Sukr framework using a mixed-method approach that includes interviewing experts in the fields of medical practice, psychology, and gamification, as well as a questionnaire for patients with diabetes in Saudi Arabia.

## Methods

A mixed-method approach was chosen for validating the framework. In particular, we used expert interviews and patient questionnaires. The interviews were conducted with a group of diabetes doctors and educators, psychiatrists and psychologists, and game experts. The questionnaire was answered by diabetic individuals from Saudi Arabia.

Ethical approvals for both studies were obtained from the Ethics Committee at the university of Southampton prior to conducting the interviews and questionnaires (reference numbers: 14208 and 15296).

### Expert Interviews

The interviews were conducted with diabetes doctors, psychologists and psychiatrists, and game experts. The doctors, psychologists, and psychiatrists were selected from public and private hospitals in Riyadh, Saudi Arabia. Those experts were chosen based on their experience with the diabetic community in Saudi Arabia and their expertise in this area. Finally, the game experts were selected from the University of Southampton. The overall total number of experts was eight (Table 1), and no more expert interviews were required since the data from the interviews approached a saturation level.

**Table 1:** Interview experts.

Expert	Expert #
Diabetic doctors	Experts #1, #3, #4, #8
Psychologists and psychiatrists	Experts #2, #5
Game experts	Experts #6, #7

A thematic analysis approach was used in the analysis of the interviews. The interviews were coded with tags that represent the eight themes of the Wheel of Sukr: self-management, self-representation, fun, growth, sustainability, motivation, esteem, and socializing. After that, the interviews were analyzed based on the coded tags and in relation to the research question and literature.

### Questionnaire

The questionnaire consisted of two parts: multiple-choice questions and Likert-scale questions, which had five choices (strongly agree, agree, neutral, disagree, and strongly disagree), each weighted from 5 to 1 respectively. The multiple-choice questions were asked in order to gather information from participants about demographics and self-management habits. The multiple-choice part of the questionnaire is where the inclusion and exclusion criteria are specified. In particular, the target population is diabetics ranging in age from 18 and 40. The second part of the questionnaire was used to measure the attitude of the participants toward the framework themes. In particular, the questions were designed to capture the overall idea of the framework and its themes instead of focusing on the technical concepts of the elements. This is because gamification may not be a familiar topic to most participants.

The target audience of the questionnaire is diabetics in Saudi Arabia, where diabetes is wide spread (3.4 million in 2015) and has a high prevalence [34,35]. Saudi Arabia is one of the top 10 countries for number of children with type 1 diabetes—it has 16,200 children 15 years old and younger in 2015, which is a quarter of the region’s total of 60,700 patients [34].

The sample contains 42 participants. The questionnaire was sent to a number of diabetes accounts on Twitter and Facebook, and participants were asked to answer and share it with other diabetics.

The Likert-scale part of the questionnaire was analyzed using Excel and Mathematica. A normality test was conducted and showed that the data do not follow a normal distribution. Therefore, a non-parametric test was used, specifically the Mann-Whitney test (1-tailed). The reference median (median of the weights) was 3, and the alpha level was specified to alpha=.05. The following hypotheses were used to test the framework themes:

- H_0_ (null hypothesis): median ≤3
- H_1_ (alternative hypothesis): median ˃3

To reject the null hypothesis, the *P* value must be *P* ≤.05. Since the questionnaire contains 34 questions, the Bonferroni correction was used [36]. Specifically, alpha is corrected to be .05/34=.0014705. Hence, the *P* value for each question should be less or equal to the new corrected value for the null hypothesis to be rejected. However, it is worth mentioning that the Bonferroni correction can be too conservative.

To ensure the clarity and validity of the questionnaire, we conducted a pilot study in which the questionnaire was given to a number of researchers for feedback. Moreover, the questions were placed randomly (not according to the themes), and a few questions were repeated in different places to ensure validity of the answers. Finally, Cronbach alpha was applied to check the internal consistency of the questionnaire.

### Sample

The snowball method was used to select the sample for the patients’ questionnaire. Prior to the distribution of the questionnaire, the sample size was estimated using the program G*power, setting alpha=.05, β=0.2, and effect size *d*=0.8 [37]. Based on this, the minimum sample size is 15. However, after distributing the questionnaire, 42 patients participated in the study, which is larger than the minimum sample size but also meets the practical application of the central limit theorem, to represent the mean of the population.

## Results

This section is divided into two subsections. The first presents the findings of the expert interviews and the second presents the results of the questionnaire.

### Expert Interview Findings

The diabetes doctors and educators provided valuable information based on their immediate experience with diabetics in Saudi Arabia. Similarly, psychologists and psychiatrists provided insight into the psychological issues that diabetics face in Saudi Arabia that can affect their self-management. As for game experts, their input was specifically focused on the elements derived from gamification/game literature. The overall findings of the interviews provided the validation of the Wheel of Sukr.

#### Fun

The idea of creating an enjoyable experience for diabetic patients was strongly welcomed by experts. Expert #3 said, “Naturally people like to be rewarded. Thus, if this is applied to the self-management of diabetes, it would be very effective.” Moreover, Expert #3 added “it will change the view and the experience of self-management of diabetes for the patient.” Expert #5 also agreed, saying, “positive reward is enjoyable in whichever form it comes. This will help patients’ self-esteem.” Furthermore, Expert #8 who regularly participates in events for diabetic patients said, “using games, competition, and fun events has shown a positive effect on diabetic patients.” This suggests that creating a game-like experience that entertains the users could be a relevant part to improving the self-management process.

#### Socializing

The ability to share the same experiences and concerns with other diabetics can offer the social and emotional support that a diabetic patient needs. Moreover, it creates a good environment for gamification where the existence of a social community adds to the value of rewards. As Expert #2 pointed out: “In today’s world, the effects of social media on young and early adolescents is very big. In fact, it could leave a stronger impact on the patient than that of the doctor.” Therefore, the social and community aspect of the framework can be essential in providing support for diabetics and tying in all the other themes of the framework.

#### Esteem

Experts agreed that diabetes has a stigma in Saudi Arabia. The parents and family of diabetic individuals enforce this by being overprotective of their diabetic children. This affects their self-management, as Expert #8 stated, “how the patient feels about diabetes has a great effect on their self-management.” Therefore, creating competitions between peers and adding leaderboards, levels, and showing progress bars in a self-management tool could be essential in boosting users’ self-esteem. This might fulfill the need for recognition and instant positive feedback in human nature. This could result in positive change in behavior regarding the self-management of diabetes.

#### Self-Management

According to some experts, some diabetics feel stigmatized and may be shy in dealing with their condition when they are participating in social activities. In turn, this could prevent them from maintaining their daily self-management routines, for example, missing taking blood glucose test results. However, the majority of experts (especially psychiatrists) argued that some families play a negative role, which prevents diabetic patients from self-managing diabetes properly. This was supported by Expert #3 who said, “The stigma on diabetes in Saudi Arabia affects the ability of some patients to perform the daily self-management activities in public or around other people.” Therefore, a gamified self-management tool could enable patients to self-manage with ease and confidence and without embarrassment or delay. The framework themes collectively could achieve this.

#### Motivation

The majority of experts agreed that many diabetic patients lack motivation in terms of self-management. Therefore, a gamified self-management tool should take this into consideration. This is why motivation is a significant part of the framework. The diabetes doctors highlighted that there is a lack of motivation in following the right procedures for self-management and caring for oneself among some patients. Both Experts #1 and #3 said that some patients are not motivated to learn about their illness and learn self-management skills. This could be also relevant to the “growth” theme (discussed later) in which feedback and progress are essential. Expert #4 expressed that game elements and rewards could be the solution to the lack of motivation, which supports the “fun” theme discussed earlier.

#### Growth

The experts agreed that elements of the growth theme such as Feedback are essential to the self-management of diabetes. As mentioned in previous elements, there is a lack of motivation in self-management and a lack of consistency. Therefore, the growth theme is a vital part of the framework, and applying all the themes combined may help in creating new habits in self-management of diabetes and creating consistency.

#### Self-Representation

Some patients might feel that they are being blamed for not taking care of their condition, for example, if their blood glucose was higher or lower than normal. However, if the patients were in an environment where they are encouraged by other peers, this could help them improve their self-managing skills. Similarly, adolescents might be reluctant to take their doctors’ instructions responsibly, possibly because it could undermine their independence, as suggested by Expert #2. This is reinforced by the opinion of Expert #6: “If the user has a sense of control of what they are doing, they will feel that things are not imposed on them and they are the actors.” Therefore, a gamified self-management app should provide an environment for diabetic patients where they feel represented and in control. Additionally, the environment should allow them to pursue and achieve their goals regarding self-managing diabetes.

#### Sustainability

Sustainability is essential to the success of any gamification app. Additionally, maintaining the same level of engagement can result in a positive change in behavior in self-management of diabetes. Experts agreed that the use of triggers and nudge theory, which are the elements of sustainability theme, might direct users into the desired behavior for self-management.

In general, the interview results showed a consensus on the importance of the framework’s themes in self-management of Saudi diabetics. In fact, Expert #2 said that the information presented in the framework is enough to start a successful project for the self-management of diabetes. The expert continued to highlight that the first implementation of the framework in a system would show any shortcomings. Next, the system would be enhanced based on user feedback. Moreover, Expert #4 pointed out the importance of such projects for the diabetic people in Saudi, especially because of the conservative nature of the community. Thus, a gamified online system could help immensely in motivating diabetics and keeping their privacy. Additionally, Expert #5 pointed out that using game elements is great for adolescence and young users. The expert continued to highlight that, in Saudi Arabia, there are many young diabetics with overprotective parents (due to their illness). Thus, using gamification and providing them with an enjoyable experience of self-management is important.

### Questionnaire Results

#### Summary

Table 2 shows the results of Part 1 of the questionnaire, which contained multiple-choice questions on demographical questions and habits related to their self-management.

**Table 2:** Data from Part 1 of the questionnaire.

Question/answer choice		Responses, %
**Age group, years**		
	18-20	21.43
	21-25	30.95
	26-30	11.90
	31-35	14.29
	36-40	21.43
**Gender**		
	Female	76.19
	Male	23.81
**When were you diagnosed with diabetes?**		
	0-1 years	14.29
	2-4 years	26.19
	5-8 years	14.29
	9+ years	45.24
**Do you have friends with diabetes?**		
	Yes	71.43
	No	28.57
**Do you keep a log of all your daily test results?**		
	Yes	40.48
	No	59.52
**How do you log your daily test results?**		
	Manually (using a pen and paper)	66.67
	Electronically (using mobile apps, computer systems, etc)	33.33

Table 3 shows the results of the second part of the questionnaire, which contained Likert-scale questions on the themes of the framework. Each theme was associated with a number of questions to measure the attitude towards the theme (see Multimedia Appendix 1). For example, the fun theme has 5 questions related to it. The frequencies for each Likert-scale item (eg, strongly agree) were averaged. This result is shown in the first column in Table 3 (39.05%). In addition, the sum of the “strongly agree” and “agree” answers is shown in the “sum” column (75.71%). Similarly, the sum of the “disagree” and “strongly disagree” is shown. The same procedure was done for all the questions.

**Table 3:** Frequency table (results as percentages).

	Strongly agree	Agree	Sum	Neutral	Disagree	Strongly disagree	Sum	
Fun	39.05	36.67	75.71	19.52	3.81	0.95	4.76	100.00
Social	33.86	34.13	67.99	18.78	11.64	1.59	13.23	100.00
Esteem	34.92	44.44	79.37	11.11	8.73	0.79	9.52	100.00
Self-management	23.81	48.81	72.62	13.10	11.90	2.38	14.29	100.00
Motivation	38.89	38.10	76.98	11.90	8.73	2.38	11.11	100.00
Growth	43.33	44.76	88.10	8.57	2.86	0.48	3.33	100.00
Self-representation	19.05	23.81	42.86	36.51	19.05	1.59	20.63	100.00
Sustainability	27.38	41.07	68.45	20.83	10.71	0.00	10.71	100.00

It is worth mentioning that the Cronbach alpha test was applied to the questionnaire, and the result was 0.91, which indicates an internal consistency of the questionnaire.

#### Fun

Overall, when we average the frequencies of the answers to each Likert item, we find that 76% (32/42) responded by strongly agree or agree, 19% (8/42) were neutral, and 5% (2/42) strongly disagreed or disagreed. This suggests that the majority of the participants support the importance of the fun theme, since it helps them overcome boredom from the repetitive tasks and provides entertainment and encouragement. It also gives them the opportunity to be appreciated for their efforts in self-management and the opportunity to positively compete with one another.

#### Socializing

Following the same procedure as described in the fun theme, 68% (28/42) of the participants answered with strongly agree or agree, 19% (8/42) were neutral, and 13% (5/42) supported strongly disagree or disagree. This suggests that diabetics like sharing their positive results with one another and establishing new friendships with their peers. This could help them cope and live positively with their condition.

#### Esteem

The results of the averaged frequencies of the answers to the Likert scale are as follows: 79% (33/42) strongly agreed or agreed, 11% (5/42) were neutral, and 4/42 (9%) strongly disagreed or disagreed. Clearly, the majority of the participants supported this theme. It is worth recalling that the esteem theme includes progress bars, leaderboards, and reputation. So, by enabling the patients to see each other’s scores and progress, they will be encouraged to self-manage. More importantly, the patients will have the chance to support and encourage each other.

#### Self-Management

Nearly three-quarters (73%, 31/42) of participants strongly agreed or agreed, 13% (5/42) were neutral, and 14% (6/42) strongly disagreed or disagreed. In particular, the majority of participants see that that a self-management system should provide information, tips, and notification. Moreover, when participant were asked about question 18, “I only record my tests to show them to my doctor” (without the conservative Bonferroni correction), 57% (24/42) agreed or strongly agreed with this statement, which could signify a need for developing better self-management habits.

#### Motivation

Over three-quarters (76%, 32/42) of participants strongly agreed or agreed, 12% (5/42) were neutral, and 12% (5/42) strongly disagreed or disagreed. The participants supported the relevance of the motivation theme. The result indicates that the participants recognize their role in managing their condition, and they are keen to keep their illness in control.

#### Growth

A majority (88%, 37/42) of participants agreed or strongly agreed, while 3% (1/42) answered disagree or strongly disagree. This theme received much support from the participants. Recall that the growth theme combines feedback, achieving goals, and tiny habits. The participants agreed that receiving feedback regarding inputs (glucose levels, food intake, etc) is important since this enables them to self-manage their condition.

#### Self-Representation

Fewer than half (43%, 18/42) of participants agreed and strongly agreed, 36% (15/42) were neutral, and 21% (9/42) disagreed or strongly disagreed. If we consider question 27 (It is important to me to keep an eye on my health through improving my self-management skills) only, then 55% (23/42) agreed or strongly agreed, while 14% (6/42) disagreed. This indicates that online self-representation is important to a significant number of the participants, which suggests that the self-representation theme is indeed relevant.

#### Sustainability

The results showed that 68% (28/42) agreed or strongly agreed, 21% (9/42) were neutral, and 11% (5/42) disagreed or strongly disagreed. A considerably large number of participants agreed that self-management apps should be regularly updated. These updates should keep them encouraged to keep using the app, for instance, by adding more levels or challenges that keep the patients motivated to use the app and therefore continue self-managing their condition in a sustainable manner.

## Discussion

### Principal Considerations

Gamification has been receiving a great deal of attention in the health care field. It has been pointed out that there is a lack of professional criteria or guidelines to help developers in creating effective apps utilizing gamification and behavioral change theories [11]. This paper presents a validated framework for gamifying the self-management of chronic illnesses to fill a gap in the literature. The validation was carried out by a mixed-method approach, which included expert interviews as well as patient questionnaires. The findings of the interviews and results of the questionnaires support the idea of incorporating gamification in the self-management process of diabetes. Both experts and patients agreed that utilizing the combined themes of the Wheel of Sukr to create a gamified self-management tool might help achieve effective self-management and behavioral change. To our knowledge, the Wheel of Sukr is the first of its kind.

Self-management of chronic illnesses, especially in diabetes, can be turned into an engaging and enjoyable experience by the use of gamification. The results of this study support this notion and indicate that both experts and diabetic patients recognize the potential of gamification in improving self-management of diabetes significantly. In particular, experts highlighted the importance of rewards, competition, and other fun elements in creating an enjoyable and rewarding experience that could lead to positive behavioral change. This, in turn, is reinforced by the findings from the patient questionnaire as shown in the Results section.

Diabetes is a lonely illness and diabetics are more prone to depression [38]. As stated in the Introduction, being part of an online community can provide patients with the emotional and psychological support they need. This is confirmed by the results of the interviews and the questionnaire. In particular, experts emphasized the positive impact of social media and peer support on patients. Additionally, the results of the questionnaire indicate that patients would like to share positive results with their peers and establish friendships with them. This can help them overcome any negative feelings they might encounter. Furthermore, the community aspect of a gamified self-management tool could enhance the value of rewards and other elements of the framework.

Moreover, the results support the notion that creating a fun and enjoyable experience for diabetic patients could help their self-esteem. In particular, the interviews indicate that patient’s self-esteem has an effect on self-management, as one expert stated, “how the patient feels about diabetes has a great effect on their self-management”. Moreover, the use of leaderboards and creating friendly competition between peers could help in boosting users’ self-esteem. The esteem theme and its elements are also supported by the majority of participants in the questionnaire. This is because it enables them to track their progress and compare it to others. Also, it could trigger positive competition between peers in a friendly, non-judgmental environment.

In 2013, a study [39] pointed out that 73% of diabetics do not document their daily glucose tests. Our results are in line with those results since, in general, patients admitted that they only record their test results for their doctors to see. This could be because they are not aware of the importance of self-management or find the self-management process mundane. The results of both the interviews and the questionnaires suggest that a gamified self-management system might help them document daily. Therefore, gamification in self-management is expected to make the self-management experience less mundane.

Furthermore, the results show that patients are willing to learn more about their condition and manage themselves better. However, this could be prevented by the shortage of well-constructed self-management tools (especially in Saudi Arabia where the patients are from). This is supported by the findings of the expert interviews that indicated that many patients are not motivated to learn or self-manage. Gamifying self-management could increase patients’ motivation. Yet, many existing gamified applications and services focus only on extrinsic motivation [40]. However, it is known that extrinsic motivation solely does not create a sustainable gamification affect [41]. The Wheel of Sukr considers both types of motivation (intrinsic and extrinsic). This allows it to address some of the issues raised by the experts and enables it to satisfy the patients’ expectations.

Providing real-time feedback that is meaningful and relevant to users is an essential part of gamification [21]. The feedback can come in many forms including rewards and graphs of blood tests. The latter will help users learn more about their condition and recognize patterns. This is supported by the majority of participants in the questionnaire who mentioned that they would like to receive feedback regarding their self-managing progress and be notified when their blood glucose fluctuates. Additionally, the importance of being represented and being autonomous were highlighted by the results of the expert interviews. Participants in the questionnaire also supported this. Many of them stressed that they would like their virtual accounts to reflect their personality, which is an integral part of the Wheel of Sukr.

The correlation between gamification and health behavior theories has been discussed [11]. It was mentioned that even though gamification apps for health and fitness do use motivation from the health behavior theory, the use of capacity or triggers is ignored. Patients mentioned the importance of keeping apps updated to sustain their interest in using them. They also noted the need to be encouraged to keep recording daily. This can be done through using triggers, which are an essential part of the framework. Moreover, patients stated that they enjoy being challenged at a level that suits their abilities. The Wheel of Sukr framework answers this by considering the user’s ability and employing triggers.

It is worth mentioning that using the mixed-method design provided a clear image of the issue [42]. It created a balance between the weaknesses of qualitative (interviews) and quantitative (questionnaire) methods [42], which allows for a well-rounded representation [43]. Moreover, the data were collected from both experts and patients, using interviews and questionnaires. The results from the interviews and the questionnaires complement each other. In fact, relying on the expert interviews alone would have resulted in a loss of all the important information provided by the patients and vice versa.

### Conclusion

This research introduced the Wheel of Sukr, which is a framework that gamifies self-management of chronic illnesses. It establishes the importance of combining gamification, behavioral theories, and standard self-management techniques to create a successful gamified self-management tool. The results are based on the input of medical doctors, psychologist/psychiatrists, and gamification researchers, and the input of patients living with diabetes. The results of the statistical analysis of the questionnaire validated the themes of the framework.

This framework can be used as a guide to help developers in creating better gamified self-managing tools by taking into account all of its themes: self-monitoring, socializing, self-representation, fun, esteem, motivation, sustainability, and growth. Each one of the themes has a number of elements. These themes combined could create the right conditions for a gamified environment to improve the self-management of chronic illnesses and make it an easier and enjoyable process.

Overall, this study suggests a general acceptance of the notion of gamifying self-management of diabetes and that it could be important in improving the experience of patients. It also supports the view that there is a need to change or enhance the current view of self-management of diabetes. The use of gamification in health care and specifically patient self-care is an important research area that needs further investigation. This framework sets the stage for further studies such as creating specific guidelines for gamification (such work is already in progress).

## Appendix

Multimedia Appendix 1 Analysis of the questionnaire.
