# Supplementary material for: Gamifying Self-Management of Chronic Illnesses: A Mixed-Methods Study
Source: JMIR Serious Games. 2016 Sep 9;4(2):e14. doi: 10.2196/games.5943 (PMC5035381; doi:10.2196/games.5943)
Supplement: Multimedia Appendix 1 [file games_v4i2e14_app1.pdf]

Appendix A

The table below shows the questions, median, and *P* value. The themes are shown as follows: Fun (F), Socializing (So), Esteem (E), Self-management (S-M), Motivation (M), Growth (G), Self-representation (S-R), and Sustainability (Su).

Analysis of The Questionnaire

	Q	**Question/s**	**M**	**P**
F	1	It is important for me when I use an App for diabetes that it is not boring in its design and usage.	4	<.001
	2	The best social networks are the ones containing entertainment features.	4	<.001
	3	I feel satisfied when my work in self-managing my diabetes is appreciated.	5	<.001
	4	I will work relentlessly to always keep a record of my test results if I have the opportunity to positively compete with other diabetic patients.	4	<.001
	5	The availability of different types of motivators in the diabetic social networks will encourage me to promptly record my tests.	4	<.001
S	6	I like to share my positive results with others	4	<.001
	7	I prefer to interact with diabetic patients through the internet but not in real life.	3	.334
	8	I would like to make friendships with diabetic patients.	4	<.001
	9	Communicating with diabetic friends will help me cope with diabetes.	4	<.001
	10	Social networks help me cope with diabetes.	4	<.001
	11	Being part of a diabetic-oriented social network will help me to live positively with diabetes.	4	<.001
	12	The social support that I receive from the Internet is important to me.	4	<.001
	13	It is difficult for my non-diabetic friends to understand and appreciate my situation.	4	.0476
	14	I would like to participate in a social network that enables me to communicate with diabetic patients.	4	<.001
E	15	I will be more encouraged to self-manage my diabetes if I see other patients' records\progress.	4	<.001
	16	I would like to share my diabetes tests with other diabetic friends, especially the positive results.	4	<.001
	17	I will be motivated to support others if I participate in a social network for diabetes.	4.5	<.001
S-M	18	I only record my tests to show them to my doctor	4	.006
	19	A self-management system for diabetes is beneficial if it provides feedback to the users based on their entries.	4	<.001
M	20	I lack self-motivation to keep a record and follow up my situation in a daily basis.	3	.102

	21	I am well aware of the importance of my role in managing my diabetes.	5	<.001
	22	I would like to self-manage my condition as best as possible along with keeping my routinely meetings with the doctor.	4	<.001
G	23	I would like to receive notifications every time I record my test results, and for them to be compared to my previous tests.	4	<.001
	24	I would like to be notified with my glucose level fluctuates abnormally.	5	<.001
	25	My understanding of how to self-manage my situation makes me feel secure and content.	4	<.001
	26	It is important to me to control my health through improving my self-management skills.	4	<.001
	27	When I use a new App, it is important for me that it helps me achieve my goals that are related to my situation. (E.g. achieving a certain level of glucose, or burning a certain number of calories.)	4	<.001
S-R	28	I would like my virtual account on the Internet (profile, photo, etc.) to reflect my personality.	4	<.001
	29	I do not use Arabic Apps (mobile or computer) because of the bad design and quality.	3	.148
	30	Diabetes is negatively looked at in Saudi Arabia	3	.067
Su	31	When using a diabetes management App, it is important that it is regularly updated.	4	<.001
	32	I need to be encouraged in order to keep recording my test on a daily basis.	4	<.001
	33	I feel bored when using an app for several times.	3	.185
	34	I enjoy being challenged at a level that suits me when I play in my mobile or computer	4	<.001
